# An Immune-Related Prognostic Classifier Is Associated with Diffuse Large B Cell Lymphoma Microenvironment

**DOI:** 10.1155/2021/5564568

**Published:** 2021-06-08

**Authors:** Xiao-Jie Liang, Rui-ying Fu, He-nan Wang, Jing Yang, Na Yao, Xin-di Liu, Liang Wang

**Affiliations:** ^1^First Clinical Medical College of Southern Medical University, Nanfang Hospital of Southern Medical University, Guangzhou 510515, China; ^2^Department of Hematology, Beijing Tongren Hospital, Capital Medical University, Beijing 100730, China; ^3^Beijing Advanced Innovation Center for Big Data-Based Precision Medicine, Beihang University & Capital Medical University, Beijing Tongren Hospital, Beijing 100730, China

## Abstract

**Background:**

Diffuse large B cell lymphoma (DLBCL) is a life-threatening malignant tumor characterized by heterogeneous clinical, phenotypic, and molecular manifestations. Given the association between immunity and tumors, identifying a suitable immune biomarker could improve DLBCL diagnosis.

**Methods:**

We systematically searched for DLBCL gene expression microarray datasets from the GEO database. Immune-related genes (IRGs) were obtained from the ImmPort database, and 318 transcription factor (TF) targets in cancer were retrieved from the Cistrome Cancer database. An immune-related classifier for DLBCL prognosis was constructed using Cox regression and LASSO analysis. To assess differences in overall survival between the low- and high-risk groups, we analyzed the tumor microenvironment (TME) and immune infiltration in DLBCL using the ESTIMATE and CIBERSORT algorithms. WGCNA was applied to study the molecular mechanisms explaining the clinical significance of our immune-related classifier and TFs.

**Results:**

Eighteen IRGs were selected to construct the classifier. The multi-IRG classifier showed powerful predictive ability. Patients with a high-risk score had poor survival. Based on the AUC for three- and five-year survival, the classifier exhibited better predictive power than clinical data. Discrepancies in overall survival between the low- and high-risk score groups might be explained by differences in immune infiltration, TME, and transcriptional regulation.

**Conclusions:**

Our study describes a novel prognostic IRG classifier with strong predictive power in DLBCL. Our findings provide valuable guidance for further analysis of DLBCL pathogenesis and clinical treatment.

## 1. Introduction

Diffuse large B cell lymphoma (DLBCL) accounts for 30–58% of all diagnosed non-Hodgkin lymphomas. The incidence of DLBCL in Europe is approximately 3.8/100,000 per year [1–3]. DLBCL is an intricate and aggressive tumor with heterogeneous phenotypic, clinical, and molecular manifestations [4–6]. Following existing chemotherapeutic approaches, the survival rate of patients with DLBCL has increased to 50–60%; however, nearly 40% of patients do not benefit fully due to the heterogeneous nature of this malignancy [7]. Hence, there is a strong need for the identification of new biomarkers to be applied in direct clinical therapy and to improve DLBCL prognosis [8, 9]. Based on advances in gene sequencing technology and numerous gene expression profiles, the prognosis in DLBCL patients has been closely associated with the tumor microenvironment (TME) [10–12]. The TME is composed of immune cells, inflammatory mediators, mesenchymal cells, and extracellular matrix molecules [12–17]. A fraction of infiltrating immune cells influences the growth and progression of tumors, defining a patient prognosis [18, 19]. At the same time, gene expression affects a variety of immune cells infiltrated in the TME [20–22]. In recent years, a novel immunotherapy approach based on kinase inhibitors that target B cell receptor signaling as well as PD-L1 and PD-1 blockade has proven successful against DLBCL [11, 23–26]. However, only a few DLBCL patients benefited from these new therapies, whereas others exhibited little or no response. Hence, analyzing the association between immune-related genes (IRGs) and overall survival (OS) may reveal the prognostic value of IRGs and novel biomarkers. The ESTIMATE algorithm uses gene expression signatures to quantify the infiltration of stromal and immune cells in tumor samples [26]. The CIBERSORT deconvolution algorithm uses gene expression profiles to detect immune cell phenotypes in bulk tumor samples with complex cell types [27]. Based on the link between DLBCL and immune cells, our study is aimed at constructing an IRG classifier capable of predicting the outcome of DLBCL patients. ESTIMATE and CIBERSORT were employed to assess the role of the TME in DLBCL.

## 2. Materials and Methods

### 2.1. DLBCL Datasets and Preprocessing

We systematically searched for DLBCL gene expression microarray datasets from the Gene Expression Omnibus (GEO, https://www.ncbi.nlm.nih.gov/geo/) database of publicly available clinical annotations. Illumina gene expression profiles were obtained using Illumina HumanRef-8 WG-DASL v3.0 for one cohort of samples (GSE32918), and Affymetrix gene expression profiles based on Affymetrix Human Genome U133 Plus 2.0 (HG-U133 Plus_2.0) were obtained for two cohorts (GSE10846 and GSE31312). The following steps were applied for dataset screening. (i) The raw CEL files from Affymetrix datasets were subjected to the robust multiarray average algorithm in Affy software [28] to perform background correction and quantile normalization. Moreover, oligonucleotides per transcript were summed up with the median polish algorithm [29]. The Illumina matrix files were subjected to quantile normalization using Lumi software. (ii) The HG-U133 Plus_2.0 probes were annotated using the hgu133plus2.db package. The Illumina HumanRef-8 WG-DASL v3.0 probe annotation sequences were obtained from the GPL8432 Platform (https://www.ncbi.nlm.nih.gov/geo/query/acc.cgi?acc=GPL8432). (iii) For multiple probes corresponding to the same gene, we used the genes with the largest average value. (iv) Complete gene expression profiles and follow-up information on patients were provided. As a result, 1022 DLBCL samples were selected, including 470, 140, and 412 samples obtained from the studies by Visco et al. [30], Barrans et al. [31], and Lenz et al. [12], respectively.

### 2.2. IRG Retrieval

We obtained a list of IRGs from the ImmPort database (https://www.immport.org/home). Different IRGs including chemokines, cytokines, and genes relevant to the immune response were reserved. Overlapping genes between GSE31312, GSE32918, and IRGs were selected for further study. The 470 samples from GSE31312 were selected as the training set for model development, whereas GSE32918 and GSE10846 were chosen as validation sets.

### 2.3. Univariate Cox Regression and Least Absolute Shrinkage and Selection Operator (LASSO) Analysis

To identify survival-associated IRGs from the 1170 overlapping ones, we performed univariate Cox regression analysis on continuous variables in cohorts GSE31312 and GSE32918. Only IRGs with a significant value of *P* < 0.05 were selected as survival-related IRGs. Based on 349 candidates from the GSE31312 cohort and 71 from the GSE32918 cohort, 25 overlapping genes were identified as survival-associated IRGs. The GSE31312 expression data of the 25 IRGs were integrated into LASSO regression to identify prognostic signatures. Specifically, the LASSO regression model was built into a multi-IRG-based classifier (containing 18 IRGs) to predict the OS of patients in the training set. LASSO regression was carried out using the “glmnet” package in R. To estimate the prognostic significance of the IRG classifier, we used the “survivalROC” package in R, thereby obtaining the receiver operator characteristic (ROC) curves of the three cohorts. The area under the curve (AUC) at different times was calculated and compared to validate the performance of the classifier. Patients were classified into the low- and high-risk groups by the median risk score of each series. Kaplan-Meier (KM) survival curves were used to analyze the survival of DLBCL patients.

### 2.4. Development and Validation of a Nomogram for Prognosis Prediction in DLBCL Patients

Next, we developed a clinically applicable method to predict the prognosis of individuals with DLBCL. Based on the results of multivariate Cox regression analysis for OS, we integrated the risk score and other clinicopathological covariates to build a nomogram in the GSE31312 cohort. Predictive factors included the risk score, age, sex, subtype, stage, ECOG performance status, and the number of extranodal sites. The nomogram was verified in the GSE10846 cohort using calibration and ROC curves.

### 2.5. Comparison of Infiltration of Stromal and Immune Cells between Low- and High-Risk Groups

To explore potential mechanisms underlying variations in OS between the low- and high-risk groups, we used ESTIMATE to obtain a microenvironment score for each sample of the three cohorts [26]. The difference in microenvironment between the two groups was analyzed using the Wilcox test [32]. Finally, we separately plotted KM curves for the low- and high-immune score groups in the three cohorts according to their cutoff values. The cutoff values were determined using the “survminer” package in R based on the relationship between patient OS and immune score in each independent cohort. The R package “MaxStat,” which iteratively tests all probable cutoff points to determine the value that achieves the maximum rank statistic, was applied to dichotomize the immune score and allocate patients to the low- and high-immune score groups in each cohort and thus diminish the computational batch effect.

### 2.6. Comparison of Leukocyte Subtypes between Low- and High-Risk Groups

To calculate the fraction of immune cells in the two groups of DLBCL patients, we employed the LM22 gene signature, which distinguished 22 human immune cell phenotypes, including B cells, natural killer cells, T cells, myeloid subsets, and macrophages [27]. The results were analyzed by CIBERSORT with a perm value set to 1000; patients with *P* > 0.05 were excluded from further investigation. The Wilcox test was used to analyze the difference in leukocyte subtype between the two groups. Furthermore, the correlation among the 22 leukocyte subtypes in DLBCL patients was calculated. Only correlation coefficients whose absolute value was greater than 0.15 were considered significant.

### 2.7. Weighted Correlation Network Analysis (WGCNA) and Transcription Factor (TF) Regulatory Network

To reveal the potential molecular mechanisms associated with the clinical significance of our IRG classifier, we explored the regulatory mechanisms of the 18 IRGs included in the classifier. First, the GSE31312 expression profiles of 318 TFs were subjected to WGCNA. We defined the first principal component of every gene module by its eigengenes, which we regarded as representative TFs. Gene significance (GS) represented the correlation between module memberships and clinical characteristics and was defined as the median *P* value (GS = lg*P*) of every TF. Risk score-correlated modules were defined by *P* < 0.01, and a higher GS value was selected for further study. Furthermore, to calculate the correlations between TFs of the highly related modules and our classifier IRGs, only genes with absolute values of correlation > 0.4 and *P* < 0.001 were retained and built into a network using Cytoscape. Finally, functional enrichment analyses based on gene ontology (GO) and Kyoto encyclopedia of genes and genomes (KEGG) pathways were performed to examine the potential molecular mechanism of these genes.

### 2.8. Statistical Analysis

All analyses were carried out in R version 3.6.1 and corresponding packages.

## 3. Results

### 3.1. Construction of the Prognostic Multi-IRG Classifier

**Figure 1:**
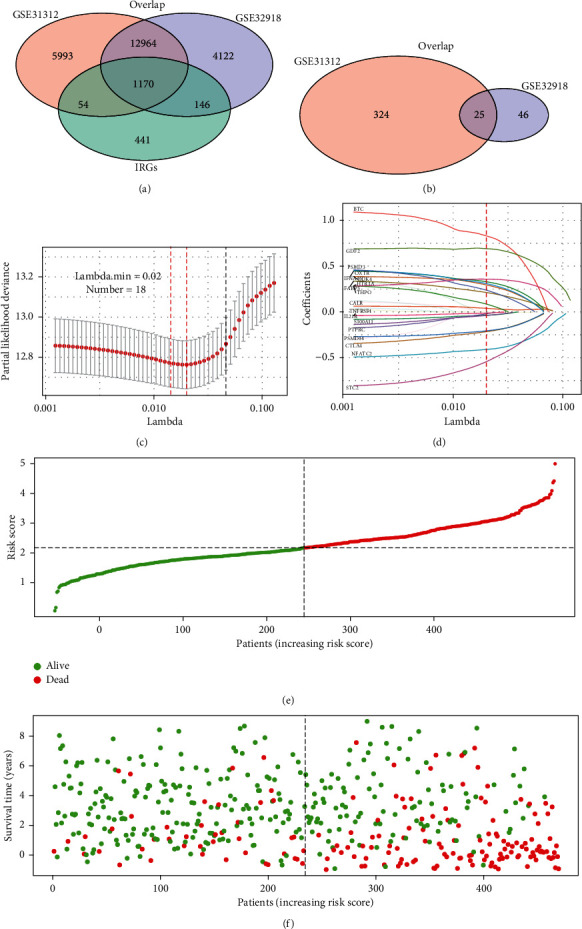
Construction of the multi-IRG prognostic classifier. (a) Venn diagram of 1170 IRGs that overlapped between GSE31312 and GSE32918 cohorts. (b) Venn diagram of 25 survival-associated IRGs that overlapped between GSE31312 and GSE32918 cohorts. (c) Eighteen IRGs selected by LASSO regression analysis. The two dotted vertical lines indicate the minimum and 1-standard error criteria employed to identify the best values. (d) LASSO coefficients of the 25 survival-associated IRGs. The red vertical line represents the best value based on the minimum criterion, which resulted in 18 nonzero coefficients. (e) Risk score distribution. (f) Survival overview of patients.

**Figure 2:**
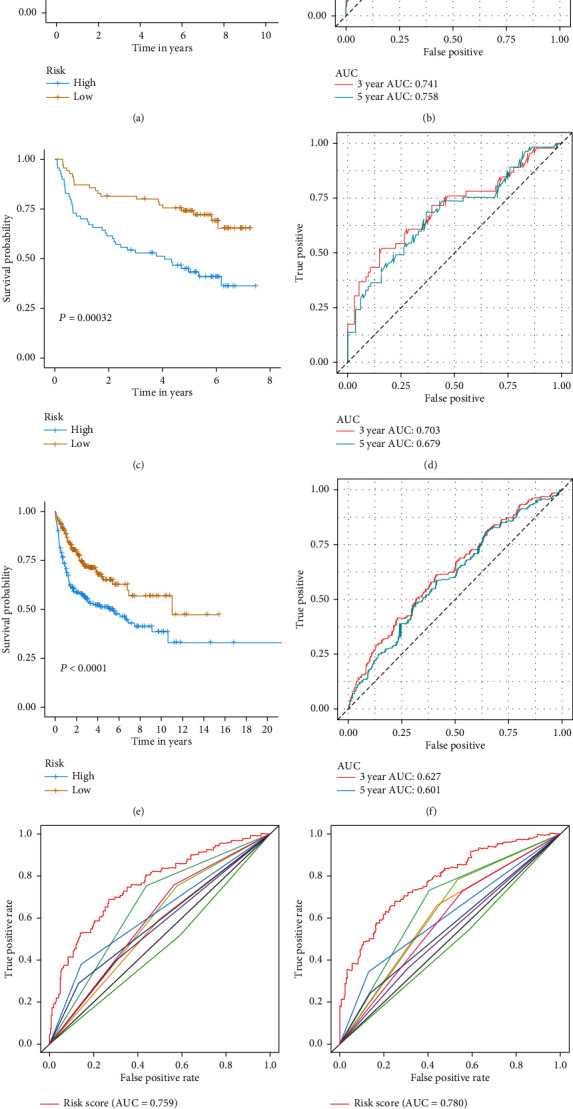
Kaplan-Meier (KM) survival curves, time-dependent ROC curves, and risk scores for patients in the training and independent external validation datasets based on the multi-IRG classifier. (a) KM survival curve of the low- and high-risk groups in the GSE31312 cohort. (b) Area under the ROC curve (AUC) of the GSE31312 cohort at three and five years. (c, d) KM survival curve and ROC curves for the GSE32918 cohort. (e, f) KM survival curve and ROC curves for the GSE10846 cohort. (g, h) Comparison of prognostic accuracy between the multi-IRG classifier and other clinicopathological characteristics in the GSE31312 cohort based on ROC curves at three (g) and five (h) years.

Univariate Cox regression analysis of the 1170 IRGs overlapping between GSE31312, GSE32918, and IRG datasets (Figure 1(a)) identified 349 IRGs from GSE31312 and 71 IRGs from GSE32918 as survival-associated IRGs based on *P* < 0.05 (Figure 1(b)). Twenty-five survival-associated IRGs were found to overlap between these two cohorts and were integrated into LASSO regression to identify prognostic biomarkers (Figures 1(c) and 1(d)). Finally, 18 IRGs were selected to build the multi-IRG classifier (Table 1). Based on the LASSO coefficients and expression levels of these 18 IRGs, we determined the median risk score for every patient in the training cohort and allocated them to either the low- or high-risk groups (Figures 1(e) and 1(f)). Based on KM survival analysis, we found that patients with high-risk scores had markedly shorter OS than those in the low-risk group (*P* < 0.0001, Figure 2(a)). To examine the prognostic significance of the classifier, we introduced ROC curves. The AUC in the training cohort was 0.741 and 0.758 for three- and five-year survival, respectively (Figure 2(b)).

### 3.2. Validation of the Multi-IRG Classifier for Survival Prediction in External Cohorts

To validate the classifier, KM survival analysis and ROC curves were generated for the GSE32918 and GSE10846 cohorts. In the GSE32918 cohort, high-risk patients tended to have shorter survival (*P* = 0.0032, Figure 2(c)), and the AUC at three and five years highlighted the predictive prognostic power of the multi-IRG classifier (Figure 2(d)). A similar result was obtained with the KM survival (*P* < 0.0001, Figure 2(e)) and ROC (Figure 2(f)) curves of the GSE10846 cohort. Furthermore, the AUC at three and five years revealed that the IRG-based classifier had better predictive power than other clinical characteristics (Figures 2(g) and 2(h)).

### 3.3. Univariate and Multivariate Cox Regression Analyses of Prognostic Factors and OS in DLBCL Patients

**Figure 3:**
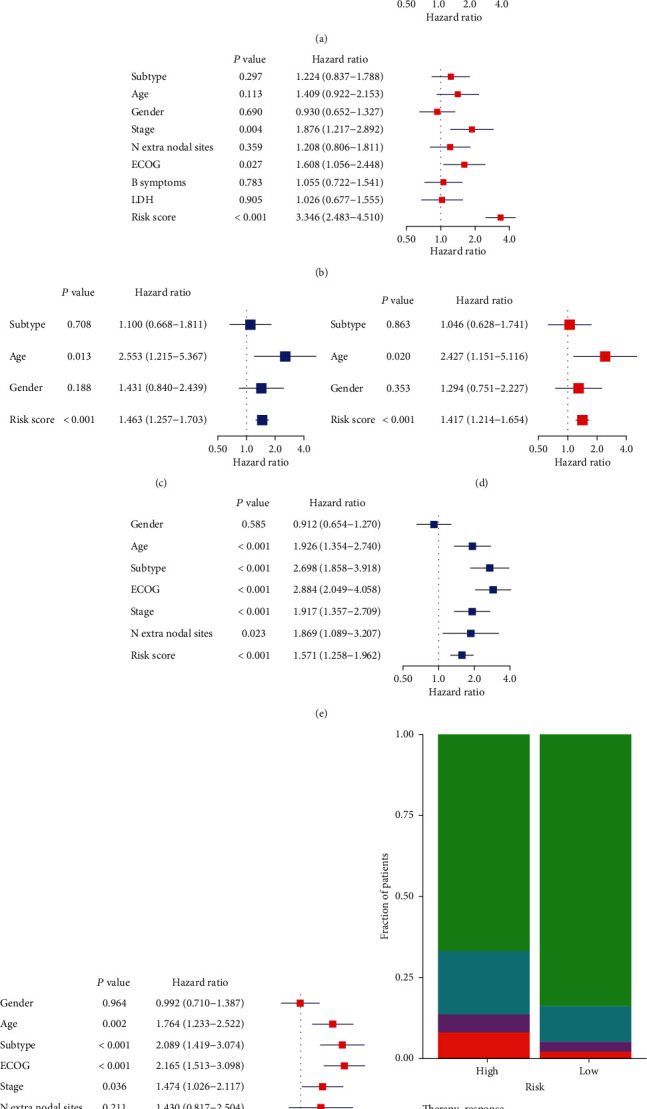
Risk score is an independent prognostic factor for DLBCL patients and may affect their chemotherapeutic response. (a, b) Forest plots of univariate and multivariate Cox regression analyses of DLBCL patients in the GSE31312 cohort. (c, d) Forest plots of univariate and multivariate Cox regression analyses of DLBCL patients in the GSE32918 cohort. (e, f) Forest plots of univariate and multivariate Cox regression analyses of DLBCL patients in the GSE10846 cohort. (g) Stacked bar plot showing the chemotherapeutic response among the low- and high-risk groups. CR: complete response; PD: progressive disease; PR: partial response; SD: stable disease.

In the GSE31312 cohort and two testing sets, we comprehensively considered clinical characteristics, such as subtype, age, gender, and stage, to perform univariate and multivariate Cox regression analyses. This allowed us to determine the significance of the multi-IRG classifier on OS. The multi-IRG classifier was found to be an independent prognostic tool for DLBCL (Figures 3(a) and 3(b), GSE31312; Figures 3(c) and 3(d), GSE32918; and Figures 3(e) and 3(f), GSE10846). A two-tailed Fisher test revealed a remarkably different chemotherapeutic response between the low- and high-risk groups. Specifically, the high-risk group tended to have a poor response and worse prognosis than the low-risk group (*P* = 0.0023, Figure 3(g)).

### 3.4. Development and Validation of a Nomogram

**Figure 4:**
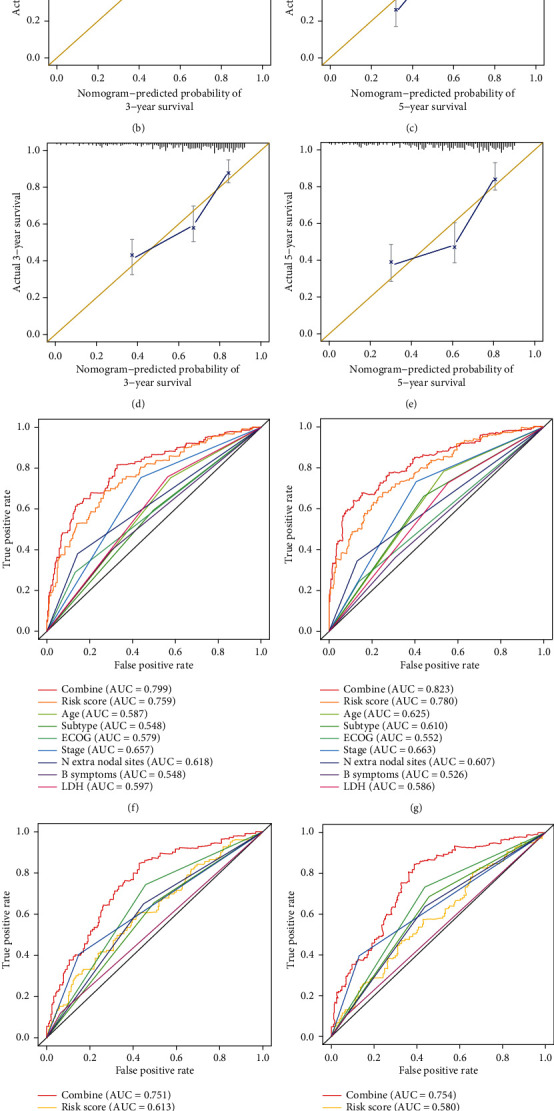
Development and validation of a nomogram. (a) Nomogram combining risk score and other clinicopathological covariates. (b, c) Calibration plots indicating the correspondence between real observations and nomogram-predicted three-year (b) and five-year (c) survival probabilities of the GSE31312 cohort. (d, e) Calibration plots indicating the correspondence between real observations and nomogram-predicted three-year (d) and five-year (e) survival probabilities of the GSE10846 cohort. (f, g) ROC curves describing the accuracy of the OS nomogram. In the training cohort, the AUC was 0.799 for three-year OS (f) and 0.823 for five-year OS (g). (h, i) ROC curves describing the accuracy of the OS nomogram. In the GSE10846 cohort, the AUC was 0.751 for three-year OS (h) and 0.754 for five-year OS (i). (j, k) ROC curves integrating age and subtype with the multi-IRG classifier to predict the outcome of DLBCL in patients from the GSE32918 cohort. The AUC was 0.719 for three-year OS (j) and 0.718 for five-year OS (k).

To predict the individual probability of OS and help clinicians provide better care to DLBCL patients, a nomogram that integrated the multi-IRG classifier and other clinical features was constructed (Figure 4(a)). A calibration curve was generated to evaluate the accuracy of the nomogram. The combined nomogram performed well in predicting the three- and five-year survival rates of patients from the GSE31312 (Figures 4(b) and 4(c)) and GSE10846 (Figures 4(d) and 4(e)) cohorts, with a prediction probability close to the observed one (Figures 4(b) and 4(e)). In the training cohort, the AUC was 0.799 and 0.823 for three- and five-year survival, respectively. The nomogram performed far better than stage, subtypes, ECOG, and other clinicopathological features when determining a patient's prognosis (Figures 4(f)–4(i)). Even when limited information about the GSE32918 cohort, such as age and subtype, was combined with our multi-IRG classifier, the prognostic accuracy of DLBCL was substantially improved (Figures 4(j) and 4(k)). Thus, the multi-IRG classifier provided additional prognostic value to existing clinicopathological predictors of DLBCL.

### 3.5. Different Immune Infiltration Scores and Leukocyte Subtypes Define Low- and High-Risk Groups

**Figure 5:**
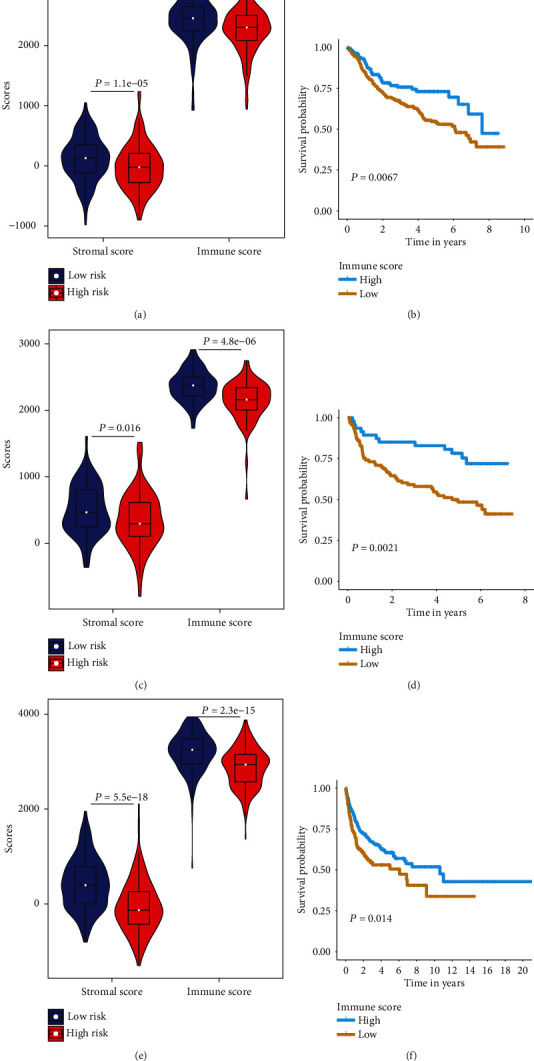
TME and immune infiltration in DLBCL patients. Upper panels: violin pots comparing the immune and stromal scores by the Wilcox test for low- and high-risk DLBCL patients. The white points represent mean values. Lower panels: KM curves of the low- and high-immune score groups in the three cohorts: (a, b) GSE31312 cohort; (c, d) GSE32918 cohort; (e, f) GSE10846 cohort.

**Figure 6:**
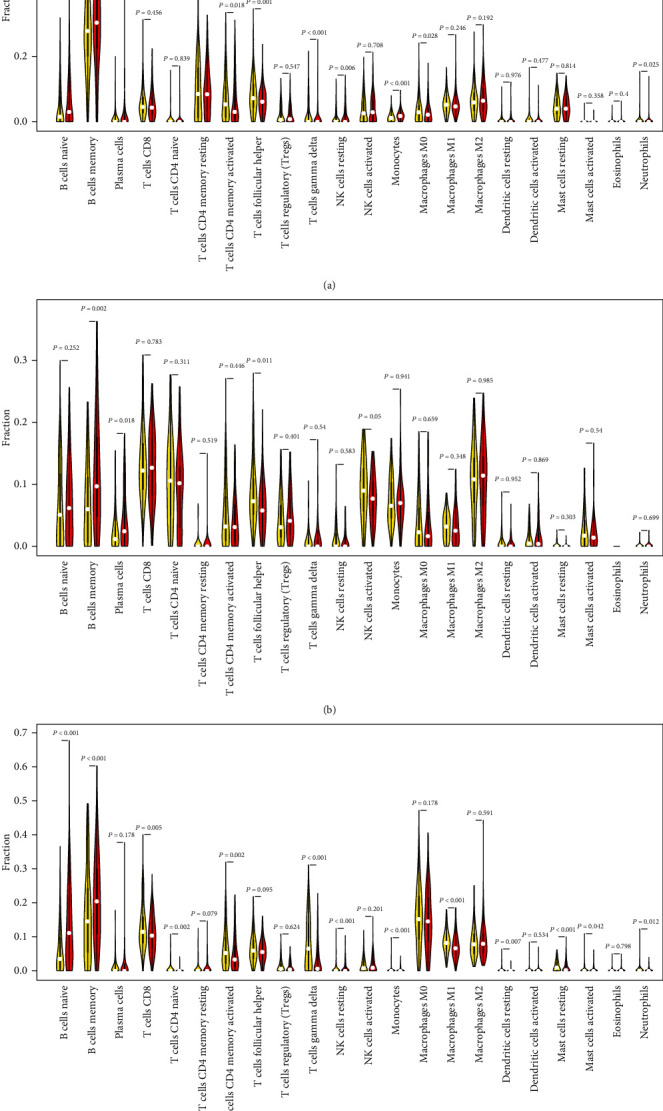
Violin plots comparing the infiltration of 22 leukocyte subtypes in low- and high-risk DLBCL samples by the Wilcox test. The white points represent mean values: (a) GSE31312 cohort; (b) GSE32918 cohort; (c) GSE10846 cohort.

We introduced the ESTIMATE algorithm to determine the immune and stromal scores of the three cohorts (Figure 5). In the training cohort, the high-risk score group tended to have a lower immune infiltration score than the low-risk group (Wilcox test *P* = 1.1*e* − 07, Figure 5(a)). The results were consistent with the GSE32918 (Wilcox test *P* = 4.8*e* − 06, Figure 5(c)) and GSE10846 (Wilcox test *P* = 2.3*e* − 15, Figure 5(e)) cohorts. Based on the cutoff values of the immune score, we separately performed KM survival analysis on the low- and high-immune score groups (Figure 5(b), GSE31312; Figure 5(d), GSE32918; and Figure 5(f), GSE10846). The results confirmed a reduced survival in the low-immune score group compared to the high-immune score group, thus indirectly verifying the poor prognosis of the high-risk score group. Next, the CIBERSORT algorithm was applied to evaluate the proportions of 22 leukocyte subtypes in the three DLBCL cohorts (Figure 6). The low- and high-risk score groups consisted of distinct immune cell types. Memory B cells, naïve B cells, CD4 memory-activated T cells, CD8 T cells, follicular helper T cells, and M2 macrophages accounted for a considerable proportion of the DLBCL immune cell infiltration.

### 3.6. Associations among TME Components in DLBCL Patients

**Figure 7:**
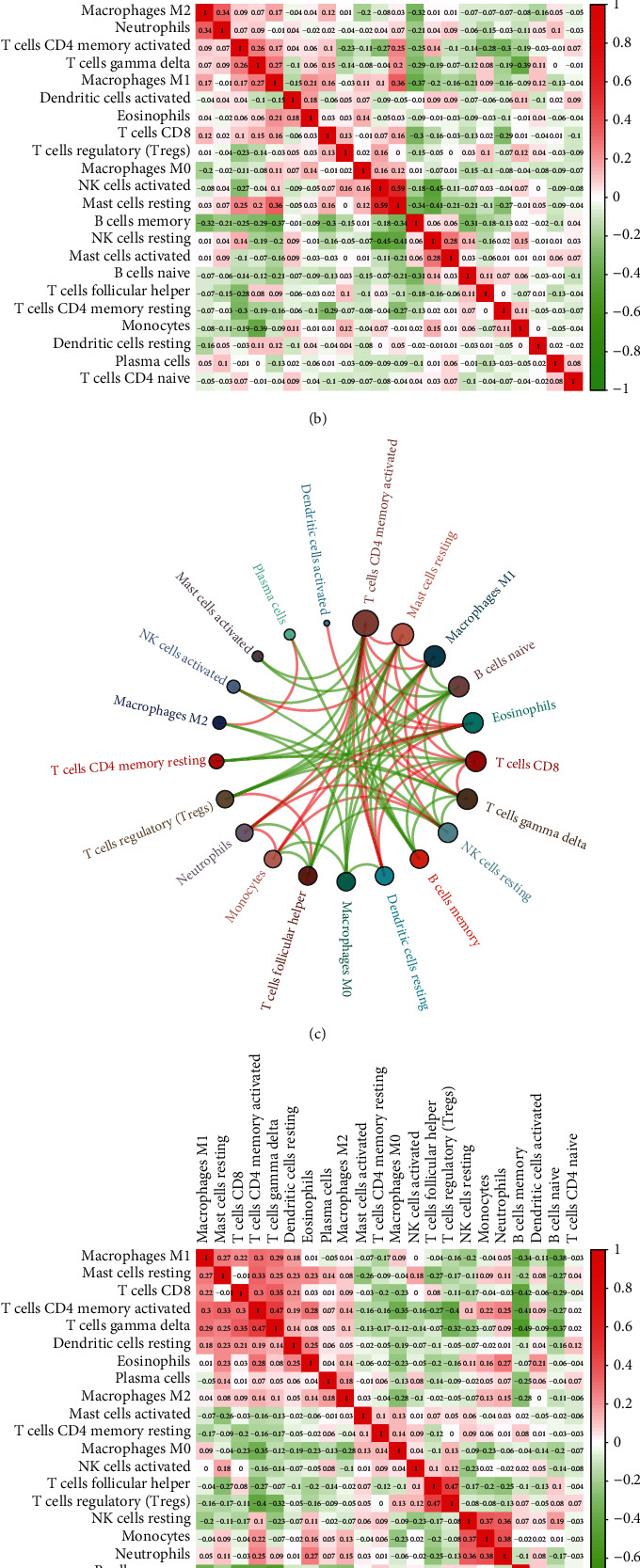
Potential connections among TME components in DLBCL patients. (a, c) Chord diagram of the correlation among 22 leukocyte subtypes in patients from the GSE31312 cohort (a) and GSE10846 cohort (c). The red edge indicates a positive correlation between the two cells, while the green one indicates a negative correlation, and node size indicates the number of cells interacting with the designated cell. (b, d) Heatmap of the correlation among 22 leukocyte subtypes in patients from the GSE31312 cohort (b) and GSE10846 cohort (d). (e) Heatmap of 22 immune cell proportions and immune infiltration, including immune and stromal scores.

To explore the potential associations among TME components in DLBCL patients, we performed correlation tests on 22 infiltrating immune cells in DLBCL (Figure 7). In the GSE31312 and GSE10846 cohorts, M1 macrophages, memory B cells, resting mast cells, CD4 memory-activated T cells, resting NK cells, CD8 T cells, and gamma delta T cells were at the core of the correlation network (Figures 7(a) and 7(c)). The correlation heatmap suggested that CD4 memory-activated T cells correlated positively with gamma delta T cells and negatively with memory B cells (Figures 7(b) and 7(d)). Additionally, there was a strong positive correlation between resting mast cells and activated NK cells. However, resting mast cells correlated negatively with memory B cells (Figures 7(a)–7(d)). Combining the ESTIMATE and CIBERSORT algorithms, we found that the high-immune cell infiltration group in DLBCL presented higher immune and stromal scores, but lower tumor purity than the low-immune cell infiltration group (Figure 7(e)).

### 3.7. TF Regulatory Network

**Figure 8:**
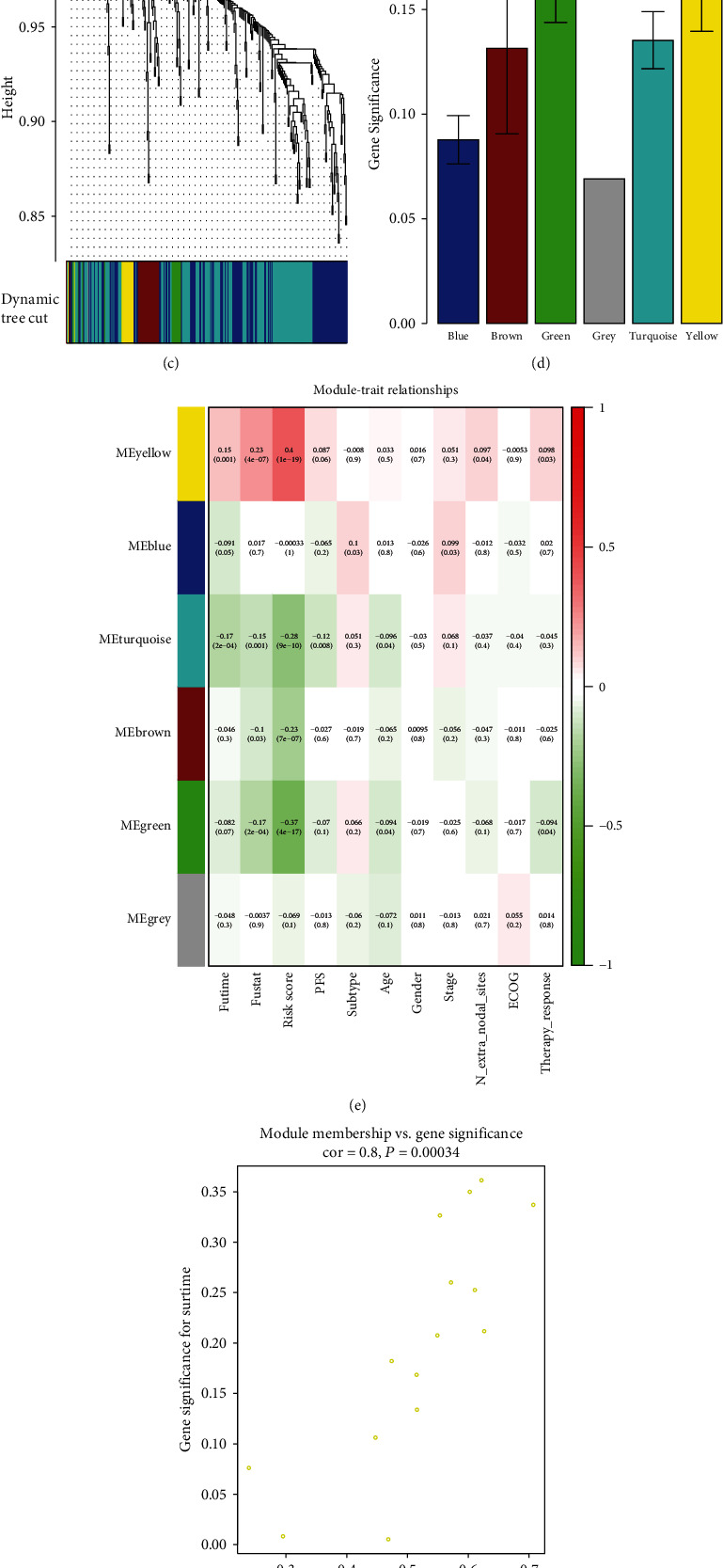
WGCNA on TFs of the GSE31312 cohort. (a) Left panel: scale-free topology fit index for soft-thresholding powers. Right panel: mean connectivity for soft-thresholding powers. (b) Scale-free *R*^2^ (*R*^2^ = 0.93). (c) Clustering dendrogram of TFs in DLBCL patients. (d) Distribution of average gene significances and errors in six modules related to risk scores. (e) Pertinence between clinical traits and six modules. (f) Scatter plots of GS for risk scores corresponding to membership in yellow, green, and turquoise modules, with their correlation coefficients and *P* value.

**Figure 9:**
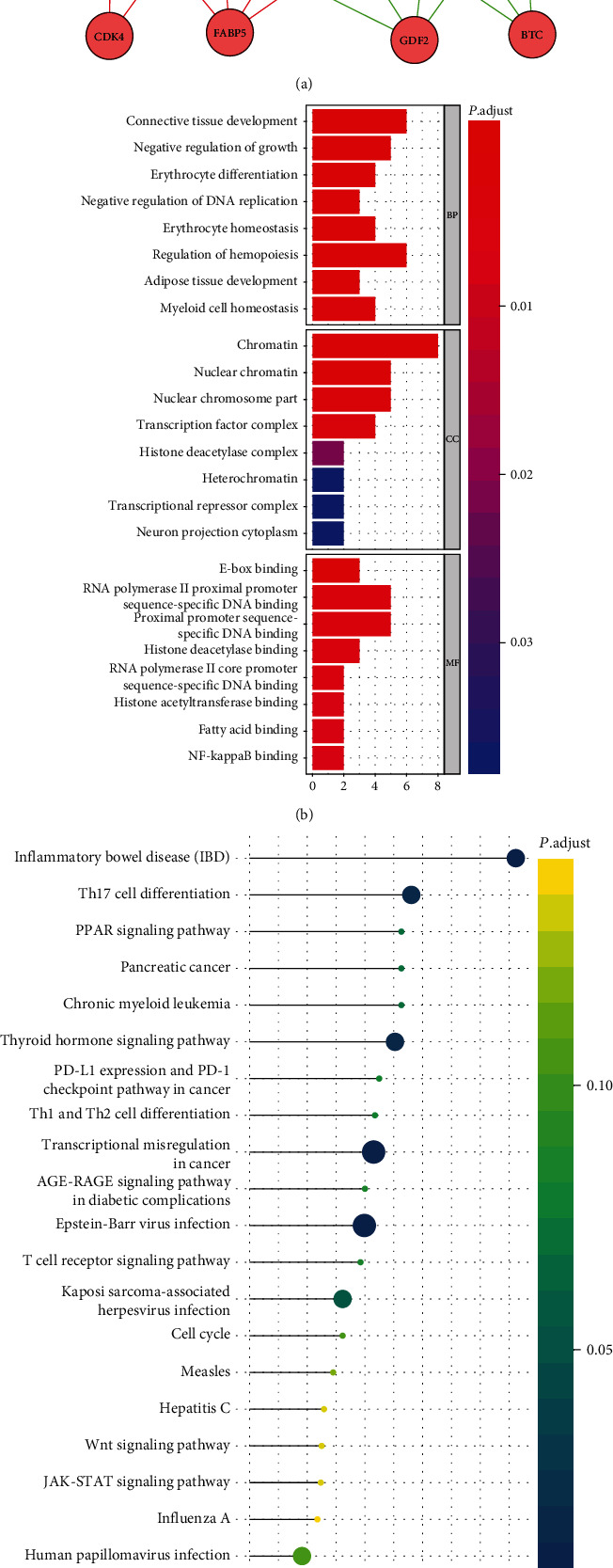
Regulatory network analysis. (a) Regulatory network diagram of risk score-relevant TFs and classifier IRGs. The green circular nodes represent protective genes (HR < 1), the red circular nodes represent dangerous genes (HR > 1), and the triangular nodes represent TFs. The red edge indicates a positive correlation, while the green one indicates a negative correlation. (b) GO analysis of the genes in the regulatory network. (c) KEGG analysis of the genes in the regulatory network.

TFs were integrated into WGCNA to explore the potential regulatory mechanisms related to the clinical significance revealed by the multi-IRG classifier. Based on the scale-free *R*^2^ (*R*^2^ = 0.93) and average linkage hierarchical clustering, we determined the soft-thresholding power and identified six TF modules (Figures 8(a)–8(c)). The yellow module, containing 15 TFs, exhibited the strongest association with the risk score of DLBCL patients. The green and turquoise modules, containing 14 and 141 TFs, respectively, were also closely connected with the patient risk score (Figures 8(d)–8(h)). TFs of the three modules were correlated with the 18 IRGs used to construct the classifier. Based on filtering criteria (absolute values of correlation > 0.4 and *P* < 0.001), we built a regulatory network in Cytoscape, which clearly demonstrated the regulatory relationships among these IRGs (Figure 9(a)). *PTPRC*, *PSMD14*, *FABP5*, *GDF2*, *STC2*, *S100A11*, and *BTC* represented the seven hub genes. Functional enrichment analysis identified the following GO biological processes: regulation of hemopoiesis, connective tissue development, and regulation of growth (Figure 9(b)). The KEGG pathways were significantly enriched in transcriptional misregulation in cancer, Epstein-Barr virus infection, human papillomavirus infection, Th17 cell differentiation, thyroid hormone signaling pathway, inflammatory bowel disease, and Kaposi sarcoma-associated herpesvirus infection (Figure 9(c)).

## 4. Discussion

As the most common subtype of non-Hodgkin lymphoma, DLBCL is an aggressive and heterogeneous tumor. Substantial progress has been made in the immunotherapy of DLBCL [33–35]. Several studies have demonstrated that the TME influences growth and progress of tumor cells and is associated with patient prognosis [10, 11, 26, 36]. The identification of immune infiltrating components could improve patient prognosis and result in biomarkers for predicting the outcome of DLBCL patients receiving immunotherapy or other treatments [10, 37–40].

Here, we developed and validated a novel tool for the prognostic stratification of patients with DLBCL into the low- and high-risk groups. The proposed risk score provided additional prognostic value to existing clinicopathological predictors of DLBCL. This is the first study to demonstrate the clinical utility of the multi-IRG signature as a prognostic tool in patients with DLBCL. Moreover, we propose a prognostic nomogram that allows for individualized estimation of three- and five-year OS probability among patients. The multi-IRG classifier and the associated nomogram may improve surveillance and guide decision-making regarding the administration of adjuvant chemotherapy and treatment duration.

The present study has identified various hub IRGs associated with OS in DLBCL patients. PTPRC (also known as CD45), a member of the protein tyrosine phosphatase (PTP) family, is involved in oncogenic transformation, cell growth, and differentiation [41]. This protein is an essential regulator of T and B cell antigen receptor signaling and is associated with a variety of cancers, including multiple myeloma, acute myeloid leukemia, and Barrett's cancer [42–44]. However, its role in DLBCL remains poorly explored [45]. This study suggests that PTPRC exerts a protective effect in patients with DLBCL, possibly through transcriptional regulation. PSMD14 encodes a component of the 26S proteasome, which is involved in apoptosis, cell cycle, and DNA damage repair [46, 47]. S100A11 is a member of the S100 family of proteins and plays an important role in intracellular calcium signaling. Altered expression and rearrangements of S100A11 have been implicated in tumors [48, 49]. Chan found that S100 expressed by antigen-presenting cells in patients with DLBCL was associated with a high survival rate [40]. FABP5 belongs to the class of fatty acid-binding proteins (FABPs) and binds to retinoic acid. This binding functions as an apoptotic as well as a differentiation signal in transformed cells [50, 51]. FABP levels are elevated in carcinomas, neoplastic skin cells, and gliomas, which are highly resistant to apoptosis [52–54]. Apoptosis-resistant (BCL-2-expressing) cells are known to alter the ability of retinoic acid to induce apoptosis [55]. Therefore, exploring the association between FABP5 and BCL-2 might reveal the mechanistic role of BCL-2 in DLBCL and point to potential therapeutic biomarkers. GDF2 (also known as BMP-9), a member of the transforming growth factor *β* superfamily, promotes the proliferation and migration of cancer cells [56]. Cyclin-dependent kinase 4 (CDK4), a crucial player in cell cycle progression, is associated with DLBCL. The CDK4 inhibitor abemaciclib strongly suppresses cell proliferation and induces apoptosis in DLBCL [57]. The present results point to a strong positive correlation between CDK4 and the TFs KZF1 and NCAPG. Identifying the mechanism underlying this relationship could accelerate the development of a suitable treatment. Functional enrichment analysis indicated that these genes might be involved in various pathways related to cancer, including Epstein-Barr virus infection, human papillomavirus infection, and Th17 cell differentiation. Th17 cells may affect patient prognosis and exert antitumor immune effects during the occurrence and progression of DLBCL. These cells and related cytokines interact with other immune cells in the TME to provide direct or indirect antitumor immunity [58–61].

Assessing the immune microenvironment using the ESTIMATE algorithm, we discovered that the degree of immune infiltration influenced OS in DLBCL patients in both the training and validation cohorts. This indicated that immune infiltration was closely associated with the outcome of DLBCL. Immune cells of the TME have been proposed for the prognostic assessment of certain cancers, such as melanoma, gastric cancer, liver cancer, and DLBCL [10, 62–65].

Based on the multi-IRG classifier, DLBCL patients were allocated to the low- and high-risk score groups. KM analysis indicated that the prognosis was better in the low-risk group than in the high-risk group, in both the training and validation cohorts. The Wilcox test showed that both stromal and immune scores differed significantly between the low- and high-risk groups. Specifically, the scores were higher in the low-risk group, which was related to greater immune infiltration and a better outcome. Therefore, we believe that the immune infiltration and OS of DLBCL patients might be influenced by the comprehensive expression of these 18 IRGs.

According to the CIBERSORT algorithm, memory B cells, naïve B cells, CD4 memory-activated T cells, CD8 T cells, follicular helper T cells, and M2 macrophages were the main infiltrating immune cells in DLBCL patients of the three cohorts. The Wilcox test indicated that synthetic expression of the 18 IRGs could lead to distinct immune cell infiltration types in DLBCL patients. The proportion of CD4 memory-activated T cells and follicular helper T cells was relatively high in the low-risk group. In contrast, memory B cells and naïve B cells were relatively more abundant in the high-risk group. This discrepancy suggests that the shorter OS of the high-risk group might result from an imbalance between these four immune cell types. CD4 memory activation in T cells correlated negatively with memory B cells. We speculate that infiltrated memory B cells in the high-risk group may somehow inhibit the infiltration of CD4 memory-activated T cells, thus contributing to the different prognosis between the low- and high-risk score groups.

Finally, we integrated multiple IRGs to construct a novel prognostic classifier via LASSO regression analysis, an unprecedented approach in DLBCL. The proposed risk score could complement existing clinicopathological predictors of DLBCL. The classifier was confirmed to have good prediction performance in the validation cohorts. Notably, few studies have applied both ESTIMATE and CIBERSORT algorithms to explore immune infiltration in DLBCL. However, the lack of further experiments investigating the proportion and specific cell types of immune infiltration is a limitation of the present study.

In conclusion, the multi-IRG classifier can effectively allocate patients with DLBCL to groups with different risks. Accordingly, IRGs may complement traditional clinicopathological risk factors to generate a comprehensive prognostic tool. The proposed nomogram incorporating the risk score and existing clinical prognostic predictors might facilitate personalized follow-up and management of patients with DLBCL.

## Figures and Tables

**Table 1:** IRGs from the prognostic classifier associated with OS in the training set.

Gene	Univariate Cox regression analysis			LASSO coefficient
	HR	95% CI	*P* value	
BTC	3.1	(1.6-6.2)	0.00094	0.830224972
THPO	2.9	(1.7-4.7)	3.60*e* − 05	0.357970287
GDF2	2.6	(1.8-3.7)	6.80*e* − 08	0.690707974
IFNA16	2.5	(1.5-4.3)	0.00092	0.329279706
HTR1A	2.2	(1.2-4)	0.0091	0.085717233
PSMD3	1.8	(1.1-2.8)	0.01	0.237502741
OXTR	1.7	(1.1-2.4)	0.0097	0.309158016
CALR	1.4	(1.1-1.7)	0.0019	0.038964007
CDK4	1.4	(1.1-1.8)	0.019	0.294461221
FABP5	1.2	(1-1.4)	0.019	0.211964852
PTPRC	0.86	(0.75-0.99)	0.029	-0.021611294
IL21R	0.86	(0.75-0.99)	0.033	-0.03685637
S100A11	0.8	(0.67-0.94)	0.0082	-0.039060903
TNFRSF4	0.78	(0.65-0.94)	0.0076	-0.01261923
PSMD14	0.74	(0.64-0.87)	0.00016	-0.20684683
CTLA4	0.68	(0.5-0.92)	0.013	-0.216324726
STC2	0.67	(0.46-0.96)	0.029	-0.543558507
NFATC2	0.54	(0.41-0.71)	7.80*e* − 06	-0.410010832

HR: hazard ratio; 95% CI: 95% confidence interval.

## Data Availability

The publicly available datasets were analyzed in this study. Data sets used in this study could be downloaded from NCBI Gene Expression Omnibus (GEO, https://www.ncbi.nlm.nih.gov/geo/) under the accession numbers GSE31312, GSE10846, and GSE32918. Illumina gene expression profiles were obtained using Illumina HumanRef-8 WG-DASL v3.0 for one cohort of samples (GSE32918), and Affymetrix gene expression profiles based on Affymetrix Human Genome U133 Plus 2.0 (HG-U133 Plus_2.0) were obtained for two cohorts (GSE10846 and GSE31312). The following steps were applied for dataset screening. (i) The raw CEL files from Affymetrix datasets were subjected to the robust multiarray average algorithm in Affy software [28] to perform background correction and quantile normalization. Moreover, oligonucleotides per transcript were summed up with the median polish algorithm [29]. The Illumina matrix files were subjected to quantile normalization using Lumi software. (ii) The HG-U133 Plus_2.0 probes were annotated using the hgu133plus2.db package. The Illumina HumanRef-8 WG-DASL v3.0 probe annotation sequences were obtained from the GPL8432 Platform (https://www.ncbi.nlm.nih.gov/geo/query/acc.cgi?acc=GPL8432). (iii) For multiple probes corresponding to the same gene, we used the genes with the largest average value. (iv) Complete gene expression profiles and follow-up information on patients were provided.
